# *N*-(3,4-Difluoro­phen­yl)phthalimide

**DOI:** 10.1107/S1600536810024189

**Published:** 2010-06-26

**Authors:** Xian-Shu Fu, Xiao-Ping Yu, Wei-Min Wang, Fang Lin

**Affiliations:** a College of Life Sciences, China Jiliang University, Hangzhou 310018, People’s Republic of China

## Abstract

In the title compound, C_14_H_7_F_2_NO_2_, the phthalimide ring system is nearly planar [maximum atomic deviation = 0.028 (1) Å] and it is twisted with respect to the attached benzene ring, making a dihedral angle of 55.70 (6)°. Weak inter­molecular C—H⋯F hydrogen bonds are present in the crystal structure.

## Related literature

The title compound is an inter­mediate in the synthesis of organic electro-luminescent materials, see: Han & Kay (2005 ▶). For the synthesis, see: Valkonen *et al.* (2007 ▶); Barchin *et al.* (2002 ▶). For a related structure, see: Xu *et al.* (2006 ▶).
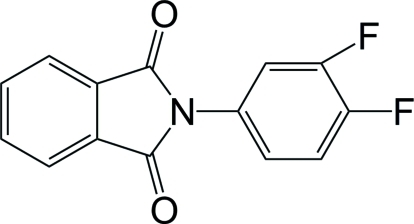


## Experimental

### 

#### Crystal data

C_14_H_7_F_2_NO_2_
*M* *_r_* = 259.21
Orthorhombic, 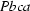

*a* = 15.101 (3) Å
*b* = 5.8093 (12) Å
*c* = 24.866 (5) Å
*V* = 2181.4 (8) Å^3^
*Z* = 8
Mo *K*α radiation
μ = 0.13 mm^−1^
*T* = 113 K
0.20 × 0.10 × 0.08 mm

#### Data collection

Rigaku Saturn CCD area-detector diffractometer
Absorption correction: multi-scan (*CrystalClear*; Rigaku, 2005 ▶) *T*_min_ = 0.975, *T*_max_ = 0.990
14468 measured reflections
1920 independent reflections
1780 reflections with *I* > 2σ(*I*)
*R*_int_ = 0.040

#### Refinement

*R*[*F* ^2^ > 2σ(*F* ^2^)] = 0.040
*wR*(*F* ^2^) = 0.115
*S* = 1.03
1920 reflections
173 parameters
H-atom parameters constrained
Δρ_max_ = 0.34 e Å^−3^
Δρ_min_ = −0.06 e Å^−3^

### 

Data collection: *CrystalClear* (Rigaku, 2005 ▶); cell refinement: *CrystalClear*; data reduction: *CrystalClear*; program(s) used to solve structure: *SHELXS97* (Sheldrick, 2008 ▶); program(s) used to refine structure: *SHELXL97* (Sheldrick, 2008 ▶); molecular graphics: *SHELXTL* (Sheldrick, 2008 ▶); software used to prepare material for publication: *SHELXTL*.

## Supplementary Material

Crystal structure: contains datablocks I, global. DOI: 10.1107/S1600536810024189/ng2786sup1.cif

Structure factors: contains datablocks I. DOI: 10.1107/S1600536810024189/ng2786Isup2.hkl

Additional supplementary materials:  crystallographic information; 3D view; checkCIF report

## Figures and Tables

**Table 1:** Hydrogen-bond geometry (Å, °)

*D*—H⋯*A*	*D*—H	H⋯*A*	*D*⋯*A*	*D*—H⋯*A*
C4—H4⋯F2^i^	0.95	2.47	3.317 (2)	149
C5—H5⋯F2^ii^	0.95	2.54	3.321 (2)	139

Symmetry codes: (i) 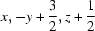; (ii) 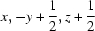.

**Fig. 1.**
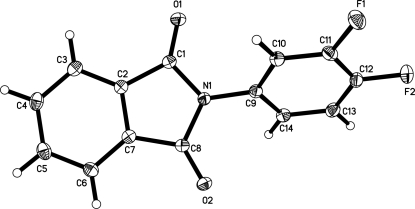
View of the molecule of showing the atom-labelling scheme. Displacement ellipsoids are drawn at the 30% probability level.

## supplementary crystallographic information

### Comment

The title compound is a key intermediate in the synthesis of organic electro-luminescent materials. The emission of light by organic molecules exposed to an electric field has been wide investigated in both an academic and industrial context. (Han & Kay, 2005).

The molecular structure of the title compound is illustrated in Fig. 1. In the title compound, the phthalimide ring system is nearly planar [maximum atomic deviation 0.028 (1) Å for N1 atom] and the dihedral angle between the benzene ring and the phthalimide plane is 55.70 (6)°, which is similar to 59.95 (4) ° found in a related compound *N*-(2-fluorophenyl)phthalimide (Xu *et al.*, 2006). Weak intermolecular C—H···F hydrogen bonding is present in the crystal structure (Table 1).

### Experimental

An acetic acid solution of phthalic anhydride (14.8 g, 100 mmol) and 3,4-difluoroaniline (9.91 ml, 100 mmol) was refluxed overnight, and then filtered. The crude product was recrystallized from ethyl acetate.

### Refinement

H atoms were positioned geometrically and refined as riding with C—H = 0.95 Å, and *U*_iso_(H) = 1.2*U*_eq_(C). The (002) and (102) reflections were omitted in the refinement.

### Crystal data

**Table:**

C_14_H_7_F_2_NO_2_	*F*(000) = 1056
*M**_r_* = 259.21	*D*_x_ = 1.579 Mg m^−^^3^
Orthorhombic, *P**b**c**a*	Mo *K*α radiation, λ = 0.71073 Å
Hall symbol: -P 2ac 2ab	Cell parameters from 5269 reflections
*a* = 15.101 (3) Å	θ = 1.6–27.9°
*b* = 5.8093 (12) Å	µ = 0.13 mm^−^^1^
*c* = 24.866 (5) Å	*T* = 113 K
*V* = 2181.4 (8) Å^3^	Prism, colorless
*Z* = 8	0.20 × 0.10 × 0.08 mm

### Data collection

**Table:**

Rigaku Saturn CCD area-detector diffractometer	1920 independent reflections
Radiation source: rotating anode	1780 reflections with *I* > 2σ(*I*)
confocal	*R*_int_ = 0.040
Detector resolution: 7.31 pixels mm^-1^	θ_max_ = 25.0°, θ_min_ = 1.6°
ω and φ scans	*h* = −17→17
Absorption correction: multi-scan (*CrystalClear*; Rigaku, 2005)	*k* = −6→6
*T*_min_ = 0.975, *T*_max_ = 0.990	*l* = −29→20
14468 measured reflections	

### Refinement

**Table:**

Refinement on *F*^2^	Secondary atom site location: difference Fourier map
Least-squares matrix: full	Hydrogen site location: inferred from neighbouring sites
*R*[*F*^2^ > 2σ(*F*^2^)] = 0.040	H-atom parameters constrained
*wR*(*F*^2^) = 0.115	*w* = 1/[σ^2^(*F*_o_^2^) + (0.0736*P*)^2^ + 0.7186*P*] where *P* = (*F*_o_^2^ + 2*F*_c_^2^)/3
*S* = 1.03	(Δ/σ)_max_ = 0.001
1920 reflections	Δρ_max_ = 0.34 e Å^−^^3^
173 parameters	Δρ_min_ = −0.06 e Å^−^^3^
0 restraints	Extinction correction: *SHELXL97* (Sheldrick, 2008), Fc^*^=kFc[1+0.001xFc^2^λ^3^/sin(2θ)]^-1/4^
Primary atom site location: structure-invariant direct methods	Extinction coefficient: 0.046 (4)

### Special details

**Table:**

Geometry. All e.s.d.'s (except the e.s.d. in the dihedral angle between two l.s. planes) are estimated using the full covariance matrix. The cell e.s.d.'s are taken into account individually in the estimation of e.s.d.'s in distances, angles and torsion angles; correlations between e.s.d.'s in cell parameters are only used when they are defined by crystal symmetry. An approximate (isotropic) treatment of cell e.s.d.'s is used for estimating e.s.d.'s involving l.s. planes.
Refinement. Refinement of *F*^2^ against ALL reflections. The weighted *R*-factor *wR* and goodness of fit *S* are based on *F*^2^, conventional *R*-factors *R* are based on *F*, with *F* set to zero for negative *F*^2^. The threshold expression of *F*^2^ > σ(*F*^2^) is used only for calculating *R*-factors(gt) *etc*. and is not relevant to the choice of reflections for refinement. *R*-factors based on *F*^2^ are statistically about twice as large as those based on *F*, and *R*- factors based on ALL data will be even larger.

### Fractional atomic coordinates and isotropic or equivalent isotropic displacement parameters (Å^2^)

**Table:**

	*x*	*y*	*z*	*U*_iso_*/*U*_eq_	
F1	0.18581 (6)	0.83152 (17)	0.21976 (4)	0.0398 (3)	
F2	0.12250 (7)	0.45784 (18)	0.17125 (4)	0.0409 (3)	
O1	0.04193 (7)	0.85824 (18)	0.40561 (4)	0.0289 (3)	
O2	0.19778 (7)	0.17995 (18)	0.40802 (4)	0.0282 (3)	
N1	0.11557 (7)	0.5119 (2)	0.39257 (5)	0.0217 (3)	
C1	0.08090 (9)	0.6931 (2)	0.42336 (6)	0.0221 (4)	
C2	0.10111 (9)	0.6332 (3)	0.48041 (6)	0.0223 (4)	
C3	0.08065 (10)	0.7479 (3)	0.52755 (6)	0.0277 (4)	
H3	0.0499	0.8905	0.5271	0.033*	
C4	0.10706 (10)	0.6458 (3)	0.57568 (6)	0.0315 (4)	
H4	0.0942	0.7206	0.6087	0.038*	
C5	0.15184 (10)	0.4367 (3)	0.57625 (6)	0.0314 (4)	
H5	0.1688	0.3708	0.6097	0.038*	
C6	0.17221 (10)	0.3224 (3)	0.52856 (6)	0.0276 (4)	
H6	0.2028	0.1794	0.5287	0.033*	
C7	0.14620 (9)	0.4251 (3)	0.48107 (6)	0.0225 (4)	
C8	0.15903 (9)	0.3464 (2)	0.42484 (6)	0.0218 (4)	
C9	0.11494 (9)	0.5016 (3)	0.33528 (6)	0.0232 (4)	
C10	0.15100 (9)	0.6824 (3)	0.30585 (6)	0.0251 (4)	
H10	0.1744	0.8147	0.3232	0.030*	
C11	0.15153 (9)	0.6626 (3)	0.25060 (6)	0.0276 (4)	
C12	0.11839 (10)	0.4696 (3)	0.22544 (6)	0.0284 (4)	
C13	0.08237 (10)	0.2919 (3)	0.25441 (6)	0.0297 (4)	
H13	0.0591	0.1601	0.2367	0.036*	
C14	0.08046 (9)	0.3082 (3)	0.31032 (6)	0.0262 (4)	
H14	0.0557	0.1874	0.3312	0.031*	

### Atomic displacement parameters (Å^2^)

**Table:**

	*U*^11^	*U*^22^	*U*^33^	*U*^12^	*U*^13^	*U*^23^
F1	0.0481 (6)	0.0388 (7)	0.0325 (6)	−0.0077 (5)	0.0028 (4)	0.0079 (4)
F2	0.0557 (7)	0.0463 (7)	0.0206 (5)	0.0059 (5)	−0.0013 (4)	−0.0034 (4)
O1	0.0296 (6)	0.0255 (6)	0.0315 (6)	0.0052 (4)	−0.0016 (4)	0.0006 (4)
O2	0.0294 (6)	0.0246 (6)	0.0305 (6)	0.0059 (4)	−0.0002 (4)	−0.0033 (4)
N1	0.0215 (6)	0.0225 (7)	0.0211 (7)	0.0022 (5)	−0.0005 (4)	−0.0009 (5)
C1	0.0179 (7)	0.0223 (8)	0.0262 (8)	−0.0003 (6)	0.0012 (5)	−0.0009 (6)
C2	0.0189 (7)	0.0235 (8)	0.0247 (8)	−0.0018 (6)	0.0008 (6)	−0.0007 (6)
C3	0.0241 (7)	0.0277 (9)	0.0313 (8)	−0.0013 (6)	0.0036 (6)	−0.0033 (7)
C4	0.0322 (8)	0.0381 (10)	0.0242 (8)	−0.0065 (7)	0.0071 (6)	−0.0039 (7)
C5	0.0334 (8)	0.0366 (10)	0.0242 (8)	−0.0064 (7)	0.0018 (6)	0.0054 (7)
C6	0.0273 (8)	0.0272 (8)	0.0282 (8)	−0.0022 (7)	0.0012 (6)	0.0048 (6)
C7	0.0198 (7)	0.0217 (8)	0.0260 (8)	−0.0027 (6)	0.0018 (6)	−0.0007 (6)
C8	0.0188 (7)	0.0208 (8)	0.0257 (8)	−0.0012 (5)	−0.0009 (6)	0.0003 (6)
C9	0.0194 (7)	0.0265 (8)	0.0236 (8)	0.0034 (6)	−0.0015 (5)	−0.0016 (6)
C10	0.0235 (7)	0.0249 (8)	0.0270 (8)	−0.0010 (6)	−0.0021 (6)	−0.0007 (6)
C11	0.0254 (7)	0.0289 (9)	0.0287 (8)	0.0021 (6)	0.0025 (6)	0.0061 (7)
C12	0.0293 (8)	0.0344 (9)	0.0217 (8)	0.0080 (7)	−0.0011 (6)	−0.0024 (6)
C13	0.0301 (8)	0.0289 (9)	0.0300 (8)	0.0032 (7)	−0.0042 (6)	−0.0070 (7)
C14	0.0248 (7)	0.0248 (8)	0.0289 (8)	0.0006 (6)	−0.0001 (6)	−0.0007 (6)

### Geometric parameters (Å, °)

**Table:**

F1—C11	1.3486 (18)	C5—C6	1.394 (2)
F2—C12	1.3507 (17)	C5—H5	0.9500
O1—C1	1.2088 (17)	C6—C7	1.380 (2)
O2—C8	1.2051 (18)	C6—H6	0.9500
N1—C1	1.4032 (18)	C7—C8	1.484 (2)
N1—C8	1.4139 (18)	C9—C14	1.385 (2)
N1—C9	1.4257 (19)	C9—C10	1.391 (2)
C1—C2	1.4923 (19)	C10—C11	1.379 (2)
C2—C3	1.383 (2)	C10—H10	0.9500
C2—C7	1.388 (2)	C11—C12	1.378 (2)
C3—C4	1.394 (2)	C12—C13	1.371 (2)
C3—H3	0.9500	C13—C14	1.394 (2)
C4—C5	1.390 (3)	C13—H13	0.9500
C4—H4	0.9500	C14—H14	0.9500
			
C1—N1—C8	111.94 (12)	C2—C7—C8	108.76 (12)
C1—N1—C9	125.04 (12)	O2—C8—N1	125.03 (13)
C8—N1—C9	122.80 (12)	O2—C8—C7	129.63 (13)
O1—C1—N1	125.29 (13)	N1—C8—C7	105.34 (12)
O1—C1—C2	129.18 (13)	C14—C9—C10	121.58 (15)
N1—C1—C2	105.51 (12)	C14—C9—N1	118.96 (14)
C3—C2—C7	121.30 (14)	C10—C9—N1	119.44 (13)
C3—C2—C1	130.35 (14)	C11—C10—C9	117.63 (14)
C7—C2—C1	108.34 (12)	C11—C10—H10	121.2
C2—C3—C4	117.29 (15)	C9—C10—H10	121.2
C2—C3—H3	121.4	F1—C11—C10	120.55 (14)
C4—C3—H3	121.4	F1—C11—C12	118.25 (14)
C5—C4—C3	121.32 (14)	C10—C11—C12	121.19 (14)
C5—C4—H4	119.3	F2—C12—C13	120.28 (14)
C3—C4—H4	119.3	F2—C12—C11	118.50 (14)
C4—C5—C6	121.00 (15)	C13—C12—C11	121.21 (14)
C4—C5—H5	119.5	C12—C13—C14	118.75 (14)
C6—C5—H5	119.5	C12—C13—H13	120.6
C7—C6—C5	117.33 (15)	C14—C13—H13	120.6
C7—C6—H6	121.3	C9—C14—C13	119.63 (14)
C5—C6—H6	121.3	C9—C14—H14	120.2
C6—C7—C2	121.76 (14)	C13—C14—H14	120.2
C6—C7—C8	129.48 (14)		
			
C8—N1—C1—O1	178.83 (13)	C9—N1—C8—C7	178.07 (12)
C9—N1—C1—O1	4.1 (2)	C6—C7—C8—O2	−3.1 (3)
C8—N1—C1—C2	−2.26 (14)	C2—C7—C8—O2	176.76 (14)
C9—N1—C1—C2	−177.01 (12)	C6—C7—C8—N1	177.27 (14)
O1—C1—C2—C3	0.4 (3)	C2—C7—C8—N1	−2.88 (15)
N1—C1—C2—C3	−178.49 (14)	C1—N1—C9—C14	−127.38 (15)
O1—C1—C2—C7	179.19 (14)	C8—N1—C9—C14	58.42 (17)
N1—C1—C2—C7	0.34 (15)	C1—N1—C9—C10	54.38 (18)
C7—C2—C3—C4	−0.1 (2)	C8—N1—C9—C10	−119.82 (15)
C1—C2—C3—C4	178.61 (14)	C14—C9—C10—C11	−0.1 (2)
C2—C3—C4—C5	−0.2 (2)	N1—C9—C10—C11	178.06 (12)
C3—C4—C5—C6	0.3 (2)	C9—C10—C11—F1	−179.79 (12)
C4—C5—C6—C7	0.0 (2)	C9—C10—C11—C12	−0.7 (2)
C5—C6—C7—C2	−0.4 (2)	F1—C11—C12—F2	0.6 (2)
C5—C6—C7—C8	179.47 (14)	C10—C11—C12—F2	−178.55 (13)
C3—C2—C7—C6	0.4 (2)	F1—C11—C12—C13	−179.79 (13)
C1—C2—C7—C6	−178.56 (13)	C10—C11—C12—C13	1.1 (2)
C3—C2—C7—C8	−179.47 (13)	F2—C12—C13—C14	179.01 (13)
C1—C2—C7—C8	1.58 (16)	C11—C12—C13—C14	−0.6 (2)
C1—N1—C8—O2	−176.48 (13)	C10—C9—C14—C13	0.6 (2)
C9—N1—C8—O2	−1.6 (2)	N1—C9—C14—C13	−177.63 (12)
C1—N1—C8—C7	3.18 (14)	C12—C13—C14—C9	−0.2 (2)

### Hydrogen-bond geometry (Å, °)

**Table:**

*D*—H···*A*	*D*—H	H···*A*	*D*···*A*	*D*—H···*A*
C4—H4···F2^i^	0.95	2.47	3.317 (2)	149
C5—H5···F2^ii^	0.95	2.54	3.321 (2)	139

Symmetry codes: (i) *x*, −*y*+3/2, *z*+1/2; (ii) *x*, −*y*+1/2, *z*+1/2.
